# Effects of exercise modality combined with moderate hypoxia on blood glucose regulation in adults with overweight

**DOI:** 10.3389/fphys.2024.1396108

**Published:** 2024-06-05

**Authors:** Chris Chow Li Tee, Mee Chee Chong, Matthew B. Cooke, Nurhamizah Rahmat, Wee Kian Yeo, Donny M. Camera

**Affiliations:** ^1^ Division of Research and Innovation, National Sports Institute of Malaysia, Kuala Lumpur, Malaysia; ^2^ Sport and Exercise Medicine Group, Swinburne University of Technology Melbourne, Hawthorn, Australia; ^3^ Sport, Performance and Nutrition Research Group, School of Allied Health, Human Services and Sport, La Trobe University, Melbourne, Australia

**Keywords:** blood glucose regulation, circulating microRNAs, exercise modalities, moderate hypoxia, overweight

## Abstract

**Purpose:** This study aimed to investigate the combined effects of moderate hypoxia with three different exercise modes on glucose regulation in healthy overweight adults.

**Methods:** Thirteen overweight males (age: 31 ± 4 years; body fat 26.3 ± 3.2%) completed three exercise trials in a randomized crossover design involving 60 min cycling exercise at 90% lactate threshold (LOW), sprint interval training (20 × 4 s all-out; SIT) and lower limb functional bodyweight exercises (8 sets of 4 × 20 s; FEX) under moderate hypoxia (FiO_2_ = 16.5%). Post-exercise oral glucose tolerance test (OGTT) was performed following each trial. Heart rate, oxygen saturation (SpO_2_), physical activity enjoyment scale (PACES), and perceptual measures were recorded during each exercise session. Venous blood was collected pre-, immediately post-, and 24 h post-exercise and analysed for plasma glucose and insulin, incremental area under curve (iAUC), and circulating microRNA expression (c-miRs-486-5p, -126-5p, and -21-5p). Interstitial glucose concentrations were measured using continuous glucose monitoring (CGM).

**Results:** Post-exercise OGTT iAUC for plasma glucose and insulin concentration were lower in SIT and LOW vs. control (*p* < 0.05) while post-exercise interstitial glucose iAUC and c-miRs were not different between exercise modes. Heart rate was greater in SIT vs. LOW and FEX, and FEX vs. LOW (*p* < 0.05), SpO_2_ was lower in SIT, while PACES was not different between exercise modes. Perceptual measures were greater in SIT vs. LOW and FEX.

**Conclusion:** Acute SIT and LOW under moderate hypoxia improved post-exercise plasma insulin compared to FEX exercises. Considering SIT was also time-efficient, well tolerated, and enjoyable for participants, this may be the preferred exercise modality for improving glucose regulation in adult males with overweight when combined with moderate hypoxia.

## Introduction

Physical inactivity is one the main causes of overweight and obesity in individuals with a body mass index (BMI) ≥ 25 and ≥30 kg⋅m^−2^ or excessive fat accumulation (>20% men and >30% women) (World Health Organization, 2021). These individuals are at greater risk of impaired metabolic homeostasis, reduced insulin sensitivity (Krogh-Madsen et al., 2010), decreased postprandial lipid metabolism (Booth et al., 2012), and loss of muscle mass (Bowden Davies et al., 2019) compared to individuals with a healthy BMI. Regular physical activity or exercise can include continuous moderate-intensity exercise (i.e., sustained and steady cycle exercise at ∼50–60% VO_2_max), high-intensity interval training (HIIT) (i.e., alternating short bursts of intense cycle exercise at >80% VO_2_max with short passive or active low-intensity recovery) (Little et al., 2014) or sprint interval training (SIT) (i.e., short bursts of “all-out” cycle effort with short passive or active low-intensity recovery) (Lei et al., 2022), and functional exercise (i.e., exercise mimic real-life activities or movements that using body weight) (Larsen et al., 2019). All exercise modes induce numerous health benefits at varying degrees including, but not limited to, improved blood glucose regulation, increased cardiovascular fitness, and beneficial anabolic (e.g., gained muscle mass) (Callahan et al., 2021) and metabolic changes (e.g., enhanced mitochondrial biogenesis and substrate metabolism) (Coffey and Hawley, 2007; Gibala and McGee, 2008; MacInnis and Gibala, 2017). Undertaking exercise on a consistent basis is therefore a central part of improving the overall health and wellbeing of individuals with overweight or obesity.

Many studies have explored varying levels of hypoxic exposure (2000–3,000 m) combined with different exercise modalities (aerobic, HIIT, and resistance exercises) (Tee et al., 2023b). Compared to normoxia, exercise performed under hypoxic conditions can further enhance adaptations to exercise (Tee et al., 2023b). Acute (i.e., single bout) (Mackenzie et al., 2011; Tee et al., 2023c) and short-term (i.e., 3 times/week for 4–6 weeks) (Haufe et al., 2008; Wiesner et al., 2010; De Groote et al., 2018) aerobic exercise combined with hypoxia (∼2,700–3,100 m) can improve metabolic health markers (e.g., plasma glucose and insulin), changes in body composition (e.g., reduced body fat percentage and increased lean muscle mass) and cardiovascular fitness in individuals with overweight and/or obese (Fernandez Menendez et al., 2018). Likewise, acute high-intensity exercise (“*all-out*” effort [20 × 6 s cycling bout]) performed under moderate to severe hypoxia (2,500–5,000 m) can improve post-exercise blood glucose levels (Lei et al., 2022). Finally, resistance exercise (50%–70% 1-RM of 4 x 4-15 repetitions) combined with moderate hypoxia (2,100–2,800 m) performed 3 times/week for 6 weeks has shown to elicit greater muscle cross-sectional area and improved glucose tolerance (Nishimura et al., 2010; De Groote et al., 2018) compared to normoxia. In contrast, several previous studies have also reported exercise performed under hypoxic conditions to not show any additive, and in some cases detrimental, effects on blood glucose or plasma insulin responses (Morishima et al., 2014; De Groote and Deldicque, 2021; Tee et al., 2023b). Despite the possible additional benefits of performing exercise under varying levels of hypoxia, there is currently no clear consensus on whether one type of exercise modality is superior to promoting metabolic adaptations associated with blood glucose regulation when the hypoxic level is similar. Findings from previous work indicate HIIT or SIT could induce positive effects on blood glucose regulation compared to aerobic exercise (Little et al., 2014). Such superiority is attributed to the greater recruitment of fast-twitch (Type II) muscle fibers (Edgett et al., 2013) and increased depletion of muscle glycogen during HIIT/SIT sessions (Vøllestad and Blom, 1985). However, no study has compared different modes of exercise to date (e.g., aerobic vs. SIT vs. functional exercises) in moderate hypoxia on blood glucose tolerance. Additionally, none of these studies have integrated continuous glucose monitoring (CGM) to provide a more comprehensive evaluation of whether different exercise modes can optimise blood glucose responses in adults with overweight.

Emerging evidence over the past 5 years has revealed the important role of a family of post-transcriptional gene repressors known as microRNAs (miRNAs) in the systemic control of metabolism within tissue (Agbu and Carthew, 2021). While the underlying mechanisms of blood glucose regulation with combined exercise and moderate hypoxia remain largely unknown, expression of specific micro-ribonucleic acids (miRNAs) may be implicated in this process (Dos Santos et al., 2022). miRNAs are short, single-stranded RNA molecules (20–30 nucleotides) that bind to specific messenger RNAs (mRNAs) and repress their corresponding protein expression (Russell and Lamon, 2015). While some forms of miRNAs reside in one or more different bodily tissues, others can be secreted into the extracellular environment in a stable form as circulating miRNAs (c-miRNAs) (e.g., plasma, serum, and urine) (Chen et al., 2008; Hanke et al., 2010). C-miRNAs such as miR-486-5p, miR-126-5p, and miR-21-5p have been implicated in mediating glucose regulation relevant to exercise (Aoi et al., 2013; Bao et al., 2018) and inflammation related to the cellular response to hypoxia (Baggish et al., 2011; Bao et al., 2018). However, very little is known regarding the effects of exercise under moderate hypoxia on the expression of such c-miRNAs and thus represents an important avenue of investigation to gain better insights into the potential mechanisms governing blood glucose regulation in response to the combined effects of exercise and moderate hypoxia.

This study primarily aimed to investigate the acute effects of moderate hypoxia combined with three different modes of exercise (e.g., aerobic, SIT, or functional exercise) on blood glucose regulation in sedentary adult males. The secondary aims of our study were to determine whether different modes of exercise combined with moderate hypoxia differently impact overall perceived discomfort, perceived breathing difficulty, leg discomfort, physical activity enjoyment, and expressions of select c-miRNAs (miR-486-5p, miR-126-5p, and miR-21-5p). We hypothesized that SIT combined with moderate hypoxia would have a superior effect on glucose regulation and alter the expression of targeted c-miRNAs in accordance with this effect on blood glucose in adult males with overweight.

## Methods

### Participants

The sample size estimation was determined from *a priori* power analysis using software G*Power (v3.1.9.7). It aimed to detect differences (effect size (η^2^_p_) = 0.28, power of 0.80, alpha of 0.05) based on a previous study comparing the effects of cycling exercise under different hypoxic severities (FiO_2_ = 20.9–14.8%, ∼0–3,000 m) on glucose area under the curve during an oral glucose tolerance test (Tee et al., 2023c). It was determined that 12 participants were needed, but 13 participants were recruited to allow for potential attrition. Thirteen males who were overweight and physically inactive (mean ± SD, age: 31 ± 4 years; height 175.0 ± 3.8 cm; body mass (BM) 80.1 ± 6.3 kg; body mass index (BMI) 26.1 ± 3.2 kg⋅m^−2^; body fat percentage (BF) 26.3% ± 3.2%; maximal oxygen consumption (VO_2_max) 24.0 ± 7.3 mL kg^−1^·min^−1^) (Table 1), participated in this study after fulfilled the eligibility criteria. Eligible participants had a BF percentage over 20%, normal blood pressure (90-120 and 60–80 mmHg systolic and diastolic, respectively), no history of cardiovascular, metabolic, or physiological diseases, were physically inactive (<150 min/week of physical activity) and had not been exposed to altitude (≥1,000 m) within 3 months prior to participation. The eligibility of participants was assessed through screening, and their levels of physical activity were evaluated using the Adult Pre-exercise Screening System (APSS) (Exercise & Sports Science Australia, 2019). This study received approval from the Human Research Ethics Committee of the National Sports Institute of Malaysia (ISNRE/A/008/2020-003/2020), Swinburne University of Technology (Australia) (20,236,622-13551) and was registered on ClinicalTrials.gov (trial identifier: NCT05627804). The study was conducted in accordance with the *Declaration of Helsinki* and written informed consent was obtained from all participants.

**TABLE 1:** Participant characteristics, blood, physiological area under the curve, and perceptual measures following a 2-h oral glucose tolerance test.

Variables	Males (*n* = 13)	Control	Low-intensity cycling (LOW)	Sprint interval training (SIT)	Functional bodyweight exercises (FEX)
*Fasting concentration*					
Fasting glucose (mmol/L)	5.1 ± 0.4	—	—	—	—
Fasting insulin (µU/mL)	33.9 ± 15.0	—	—	—	—
Systolic blood pressure (mmHg)	116 ± 4	—	—	—	—
Diastolic blood pressure (mmHg)	77 ± 6	—	—	—	—
***Physical activity/VO***_***2***_ ***max***					
Time (min/week)	88 ± 28	—	—	—	—
VO_2_max (mL·kg^−1^·min^−1^)	24.0 ± 7.3	—	—	—	—
*Blood samples and physiological measures*					
2 h OGTT plasma glucose AUC (mmol/L⋅h)	—	181 ± 48	128 ± 59	120 ± 94	147 ± 73
2 h OGTT plasma insulin AUC (µlU/mL⋅h)	—	5,419 ± 2,711	3,840 ± 1822	2,242 ± 1,472	5,315 ± 2,999
2 h interstitial glucose (mmol/L⋅120 min^-1^)	—	—	45 ± 6	44 ± 9	46 ± 8
24 h interstitial glucose (mmol/L⋅24 h)	—	—	112 ± 15	112 ± 14	117 ± 13
Heart rate (bpm)	—	—	123 ± 10	139 ± 10	131 ± 10
Arterial oxygen saturation (SpO_2_, %)	—		91 ± 1	92 ± 1	92 ± 1
*Perceptual measures (CR10)*					
Overall perceived discomfort	—	—	3 ± 1	5 ± 1	3 ± 1
Breathing difficulty	—	—	3 ± 1	5 ± 1	3 ± 1
Leg discomfort	—	—	3 ± 1	5 ± 1	3 ± 1
PACES	—	—	104 ± 12	111 ± 9	111 ± 11

Values are presented as mean ± SD.

AUC, area under the curve; FEX, functional bodyweight exercises; LOW, low-intensity cycling; OGTT, oral glucose tolerance test; PACES, physical activity enjoyment scale; SIT, sprint interval training; VO_2_max, maximal oxygen consumption.

## Experimental protocol

**FIGURE 1:**
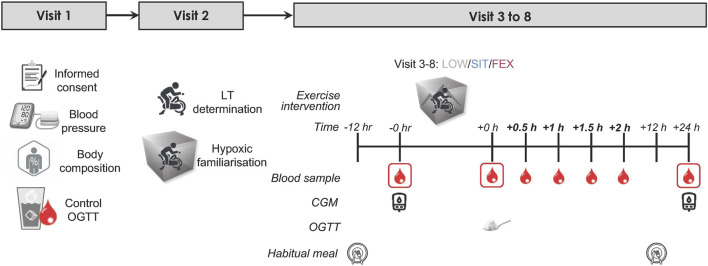
Study schematic: an overview of the entire study. Eligible healthy, overweight participants (*n* = 13) completed three exercise modes under moderate hypoxia in a randomized order separated by ≥ 7 days with 75 g of glucose dissolved in water was provided immediately following each exercise trial for the measurement of OGTT. Blood samples were collected pre, immediately post, every 0.5 h for 2 h post and post-24 h each exercise trial. OGTT, oral glucose tolerance test; LT, lactate threshold test; CGM, continuous glucose monitor.

Figure 1 provides an overview of the experimental protocol. Participants attended eight laboratory visits in total, including two baseline visits, three exercise trials, and three post-24 h follow-up visits after each respective exercise trial. Throughout the experimental period, participants were instructed to maintain their habitual diet and daily activities. Participants were instructed to attend the laboratory following an overnight fast for 10–12 h for each visit. During the first visit, measurements of blood pressure and body composition were taken, and a control (CTL) 2 h oral glucose tolerance test (OGTT) was conducted (described subsequently). Body composition parameters including body mass, height, and body fat percentage (BF) were measured using bioimpedance analysis (Inbody 770, Cerritos, CA, United States), with participants wearing light clothing.

During the second visit, participants underwent a lactate threshold (LT) test, maximal oxygen consumption (VO_2_max) test, and a familiarisation trial in an environmental chamber (Welltech Instruments, Hong Kong) without operating the chamber to allow participants to familiarize with exercising in the chamber. Participants rode a cycle ergometer (Velotron Racermate, Seattle, United States) with an initial load of 50 W. The load was increased by 15 W increments every 4 min, while participants maintained a constant pedal frequency (cadence ∼90 rpm) until reaching LT, which is defined as the power output preceding a sudden and sustained increase in lactate (≥1.0 mmol/L greater than baseline), as described previously (Farrell et al., 1979). Upon reaching LT, participants continued to pedal, and the load was increased progressively in 25W increments every 1 min until volitional fatigue. We assessed VO_2_max using a metabolic cart (Parvomedics True Max 2,400, Utah, United States) with the pulmonary gas exchange and ventilation averaged into 60-s time bins. We assumed that participants had reached VO_2_max when two or more of the following criteria were met: (i) respiratory exchange ratio >1.10, (ii) VO_2_ levelling off despite an increase in power output, and (iii) heart rate within 10 beats/min of the predicted maximum heart rate. Heart rate (HR) was recorded throughout the test using a heart rate monitor (Polar H10, Polar Electro OY, Kempele, Finland). Ratings of perceived exertion (RPE) were recorded at the end of each stage using Borg 1–10 scale. The first and second visits were scheduled at least a week before the third visit (exercise trial).

During visits three to 8, participants completed three exercise trials in a randomised crossover design. Each trial was separated by a minimum of 7 days. All exercise trials were conducted at 7:30 a.m. The exercise trials consisted of three modes: (i) low-intensity cycling (LOW), (ii) sprint interval training (SIT), and (iii) functional bodyweight exercises (FEX) under moderate hypoxia (MH; FiO_2_ = 16.5% corresponding to a simulated altitude of ∼2000 m). Low-intensity cycling involved 60-min at 90% LT. SIT involved 20 × 4 s all-out against a load equivalent to 7.5% of bodyweight on a cycle ergometer (Velotron Racermate, Seattle, United States) separated by 1-min 56 s active rest periods 30 W (total 40 min of exercise). Functional bodyweight exercises involved four 20-s rotation-based exercises (half-squat, gluteal contractions, calf raises, and knee raises) for eight sets separated by 2-min active rest (walking around the chamber) each set (total 31 min of exercise including active warm-up). The selection of each of these three different exercise modes was based on previous studies that have each been shown to exert beneficial effects on blood glucose regulation in adults with overweight along with being manageable and feasible (Dempsey et al., 2016; Lei et al., 2022; Tee et al., 2023c). Similarly, moderate hypoxia (FiO_2_ = 16.5%, ∼2000 m) was chosen based on our previous work showing this level of altitude to exert the most positive effect on blood glucose regulation compared to other levels of hypoxia (Tee et al., 2023c). All exercise trials were conducted in an environmental chamber, where the temperature and relative humidity were maintained at 20°C and 50%, respectively.

## Measurements on exercise trial days

A continuous glucose monitor sensor (CGM; FreeStyle Libre™, Abbott Diabetes Care, Witney, United Kingdom) was affixed to the back of the upper arm following the manufacturer’s instructions. Participants were instructed to scan the sensor with a CGM reader every 8 h to reduce the likelihood of missing data.

Venous blood samples were obtained from the antecubital vein via venipuncture pre-, immediately post-, and 24 h post-exercise. HR and oxygen saturation (SpO_2_) were recorded at 10-min intervals during cycling exercise. Following exercise, participants were asked to reflect on their subjective perceptions post-exercise, including overall perceived discomfort, perceived breathing difficulty, and leg discomfort, using the modified Borg CR10 scale (Tee et al., 2023a). Additionally, their enjoyment of physical activity was evaluated using the physical activity enjoyment scale (PACES) (Kendzierski and DeCarlo, 1991). Symptoms of acute mountain sickness were assessed using the Lake Louise Questionnaire (Roach et al., 2018) at the end of the exercise trial. Participants showed no symptoms of acute mountain sickness during hypoxic exposures.

### Oral glucose tolerance test (OGTT)

Immediately after exercise, a 2 h oral glucose tolerance test was performed. Participants ingested 75 g of glucose (Glucolin glucose powder) dissolved in 250 mL of water. Venous blood samples (EDTA and sodium fluoride) were drawn immediately post glucose ingestion and at 30-min intervals up to 120 min (30, 60, 90, and 120 min).

### Blood samples analysis

Upon collection, blood samples were centrifuged at 2,000 x *g* for 10 min at 4°C. Plasma glucose levels were measured using a biochemistry analyzer (YSI 2900D Biochemistry Analyzer, Yellow Springs, OH, United States) with a coefficient of variation (CV) of <2.0%. Plasma insulin levels were analysed using commercially available enzyme-linked immunosorbent assay kits (Insulin ELISA, DE2935, Demeditec, Germany. The intra-assay and inter-assay variability for insulin were <2.6% and <6.0%, respectively. The assay was conducted following the manufacturer’s instructions and was measured in duplicate.

### Circulating miRNA (c-miRNA) extraction and reverse transcription

Plasma samples stored at −80°C were thawed on ice prior to c-miRNA extraction according to the manufacturer’s instructions (Cat. No. 217204; Qiagen, VIC, Australia). Briefly, 200 µL of plasma were subsequently aliquoted into the 1.5 mL microcentrifuge tube and mixed with 60 µL of buffer RPL and 20 µL of buffer RPP (Qiagen, VIC, Australia). Samples were vortexed, incubated for 3 min, and centrifuged at 12,000 *g* for 3 min at room temperature. The supernatant was then transferred to a 1.5 mL microcentrifuge tube and mixed thoroughly with isopropanol. Samples were then transferred to RNeasy UCP MinElute columns (Qiagen; VIC, Australia) in a 2 mL collection tube and centrifuged at 8,000 *g* for 15 s at room temperature. After washing and centrifuging with RWT and RPE buffers (Qiagen; VIC, Australia), columns were washed a final time with 80% ethanol. Columns were then centrifuged with lids open to dry any residual ethanol as per the manufacturer’s instruction. For RNA elution, columns were placed in a new 1.5 mL collection tube and 14 µL of RNase-free water was added directly to the spin column membrane and centrifuged for 1 min at 20,817 *g* (full speed) at room temperature. Extracted RNA was stored at −80°C for subsequent cDNA synthesis. Extracted miRNAs were reverse transcribed in a 10 µL reaction that included reverse transcriptase and miRNA using the miRCURY LNA RT Kit (Cat. No. 339340; Qiagen, VIC, Australia) in a BioRad thermal cycler (BioRad, Australia) according to the manufacturer’s protocol. The resulting cDNA was stored at −20°C.

### Reverse transcription and real-time polymerase chain reaction (RT-PCR)

Quantification of c-miRNAs was performed on a 384-well plate BioRad™ CFX384 Touch Real-Time PCR Detection System using the Qiagen™ miRCURY LNA SYBR Green PCR Kit (Catalog no. 339346; Qiagen, Victoria, Australia) and miRCURY LNA miRNA PCR Assays (Cat. No. 339346; Qiagen, VIC, Australia) in a 10 μL reaction, following all manufacture’s protocol instructions. Three c-miRNAs were selected *a priori* from previous studies: hsa-miR-486-5p (GeneGlobe Catalog Number YP00204001), hsa-miR-21-5p (GeneGlobe Catalog Number YP00204230), and hsa-miR-126-5p (GeneGlobe Catalog Number YP00206010). These specific c-miRNAs were selected due to their previous implication in cellular processes underlying acute exercise responses. Specifically, we chose to study mediators of glucose regulation relevant to exercise (miR-486-5p and miR-126-5p) (Aoi et al., 2013; Bao et al., 2018) and inflammation related to cellular response to hypoxia (miR-21-5p) (Baggish et al., 2011; Bao et al., 2018). The GeneGlobe (www.geneglobe.qiagen.com/analyze) was used for target prediction of miRNAs altered between three-time points (pre-, immediately post-, and 24 h post-exercise) of different exercise modes under moderate hypoxia for those that were determined to be statistically significant (*p* < 0.05). Expression of target c-miRNAs was normalized to reference gene SNORD48 (GeneGlobe Catalog Number YP00203903) which itself was not different in expression between time points (*p* = 0.25) or exercise groups (*p* = 0.42). The 2^∆∆^ CT method of relative quantification was used to calculate the relative abundance of c-miRNAs (Livak and Schmittgen, 2001). Where the relative abundance of c-miRNAs in plasma was >3 SD from the mean, measures were excluded from analysis at all time points (i.e., pre, immediately post-, and 24 h post-exercise).

### Statistical analysis

The incremental area under the curve (iAUC) for venous plasma glucose and insulin concentrations during 2 h OGTT were calculated using the trapezoid method (Potteiger et al., 2002). Similarly, the total AUC (AUC_total_) for 24 h interstitial glucose derived from CGM was calculated using the trapezoid method. The normality of the data distribution was tested using the Shapiro-Wilk test, which passed, and the homoscedasticity test was used to check the variance homogeneity between groups. All data were analysed using one-way repeated-measures analysis of variance (ANOVA) to compare different exercise modes under moderate hypoxia. Physiological and perceptual measures between exercise modes under moderate hypoxia were analysed using one-way repeated-measures ANOVA. Differences in c-miRNA expression were evaluated by two-way repeated measures of ANOVA to analysed variations over time (pre, post, and 24 h post) and across exercise conditions (LOW, SIT, and FEX). Tukey-adjusted *p* values were performed if the main effect was observed. Effect sizes were described in terms of partial eta-squared (η^2^_p_, with η^2^ ≥ 0.06 representing a moderate effect and η^2^_p_ ≥ 0.14 a large effect) (Cohen, 2013). Given our sample size (*n* < 20), Hedge’s *g* effect sizes were assessed to determine meaningful differences (with *g* = 0.38–0.75 representing a moderate effect and *g* ≥ 0.76 representing large effect sizes) (Brydges, 2019). All data are expressed as mean ± SD. All statistical analyses were performed using GraphPad Prism version 9.2.0 (GraphPad Software, San Diego, CA). Statistical significance was set at the level of *p* < 0.05.

## Results

### Blood samples analysis

**FIGURE 2:**
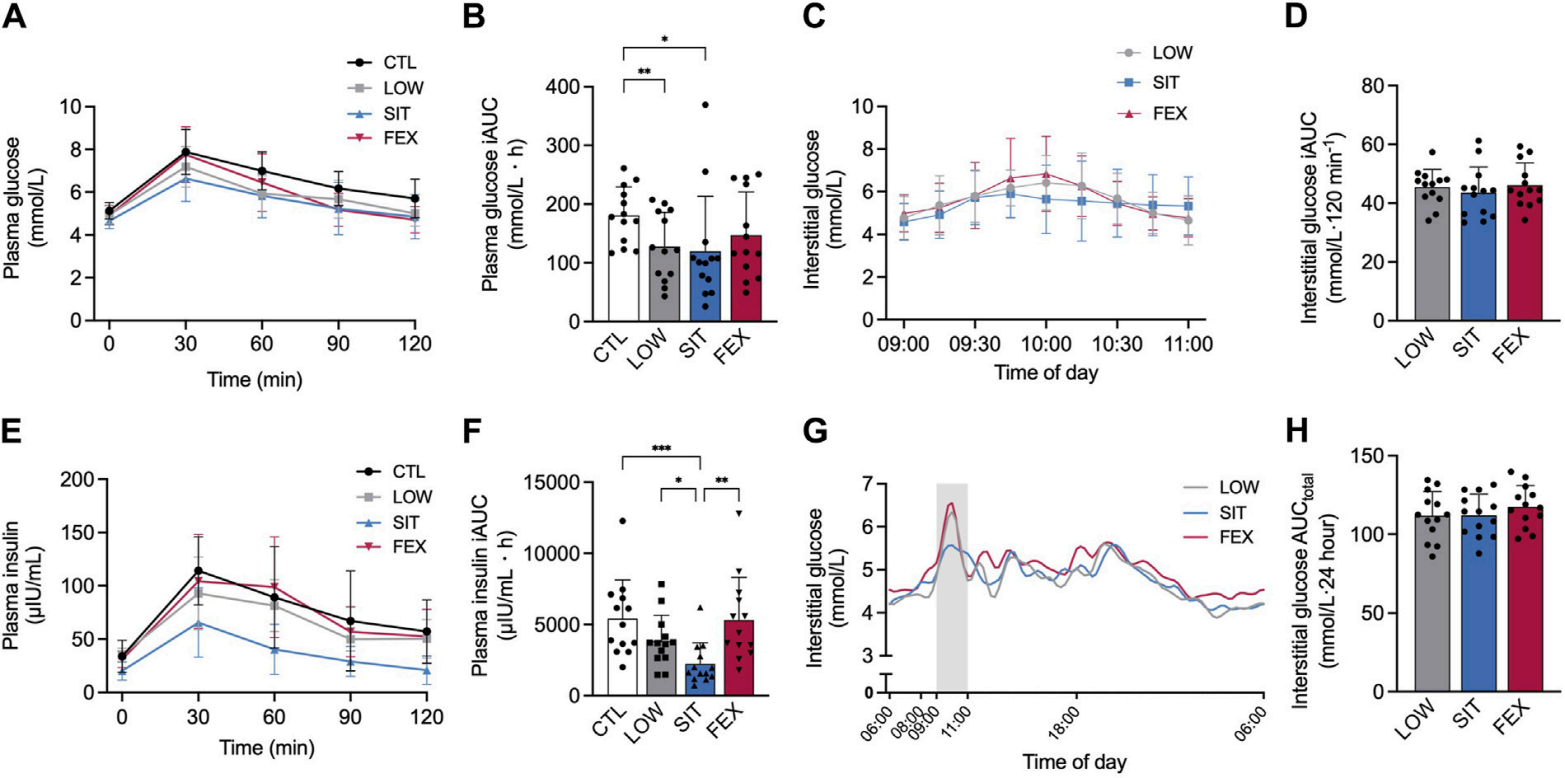
Two-hour responses following an OGTT after each of three exercise modes under moderate hypoxia for venous plasma glucose **(A)**, interstitial (CGM) glucose **(C)**, plasma insulin **(E)** concentrations and subsequent 2 h incremental AUC for plasma glucose **(B)**, plasma insulin **(F)** and interstitial glucose **(D)** concentration 24 h interstitial (CGM) glucose concentration **(G)** and CGM AUC **(H)** from 06:00 until 06:00 the following morning. Values are mean ± SD. *p < 0.05, **p < 0.01, ***p < 0.001 denotes a statistically difference between conditions. Area shaded with grey represents OGTT. AUC, area under the curve; CGM, continuous glucose monitor; CTL, control; SIT, sprint interval training; FEX, functional exercise; OGTT, oral glucose tolerance test.

There was a significant main effect of exercise conditions on plasma glucose iAUC (*p* = 0.008; η^2^_p_ = 0.32; Figure 2A). Plasma glucose iAUC was significantly lower in SIT and LOW compared to CTL (−34% ± 94%; *p* = 0.028; *g* = 0.80; −30% ± 22%; *p* = 0.006; *g* = 0.97, Figure 2B, respectively). No significant difference was observed between FEX and CTL (−19% ± 53%; *p* = 0.16; *g* = 0.53), as well as between LOW and SIT (+6 ± 59%); LOW and FEX (+16 ± 25%); or SIT and FEX (−5 ± 8%); pooled values: *p* = 0.16–0.98 and *g* = 0.27–0.98, respectively).

There was no difference between conditions for interstitial glucose iAUC during 2-h OGTT (−4 ± 44%; −6 ± 14%; pooled values: *p* = 0.61–0.68, respectively; Figures 2C,D). A main effect of exercise conditions was detected for plasma insulin iAUC (*p* = 0.003; η^2^_p_ = 0.41; Figure 2E). Plasma insulin iAUC was significantly lower in SIT compared to CTL, LOW and FEX (−59% ± 46%; *p* < 0.001; *g* = 1.41; 42% ± 19%; *p* = 0.04; *g* = 0.93; −58% ± 51%; *p* = 0.006; *g* = 1.26; Figure 2F). No significant differences were observed between LOW or FEX and CTL (−41% ± 49%; −2 ± 10%; pooled values: *p* = 0.06–0.99, respectively), as well as LOW compared to FEX (+28 ± 39%; *p* = 0.46). For interstitial glucose and 24 h AUC_total_, no significant differences were observed between SIT or FEX and LOW (0% ± 12%; 4% ± 12%; pooled values: *p* = 0.27–0.99, respectively), as well as SIT compared to FEX (4% ± 1%; *p* = 0.16; Figures 2G,H).

### Energy intake

Participants adhered to recording their 24-h dietary intake on exercise trial days, with no differences observed in macronutrients and energy intake across different exercise modes (pooled values: *p* = 0.16–0.99; Figures A and B, Supplementary Figure S1).

### Circulating-miRNA expression

**FIGURE 3:**
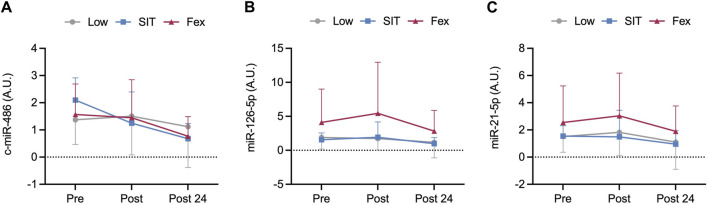
Pre, immediately post and 24 h post-exercise concentration of circulating microRNA of c-miR-486-5p **(A)**, c-miR-126-5p **(B),** and c-miR-21-5p **(C)** at three exercise modes under moderate hypoxia. Values are mean ± SD.

There were no differences in the expression of either c-miR-486-5p, c-miR-126-5p, or c-miR-21-5p post-exercise or between groups (pooled values: *p* = 0.44–0.97; Figures 3A–C).

### Physiological measures

**FIGURE 4:**
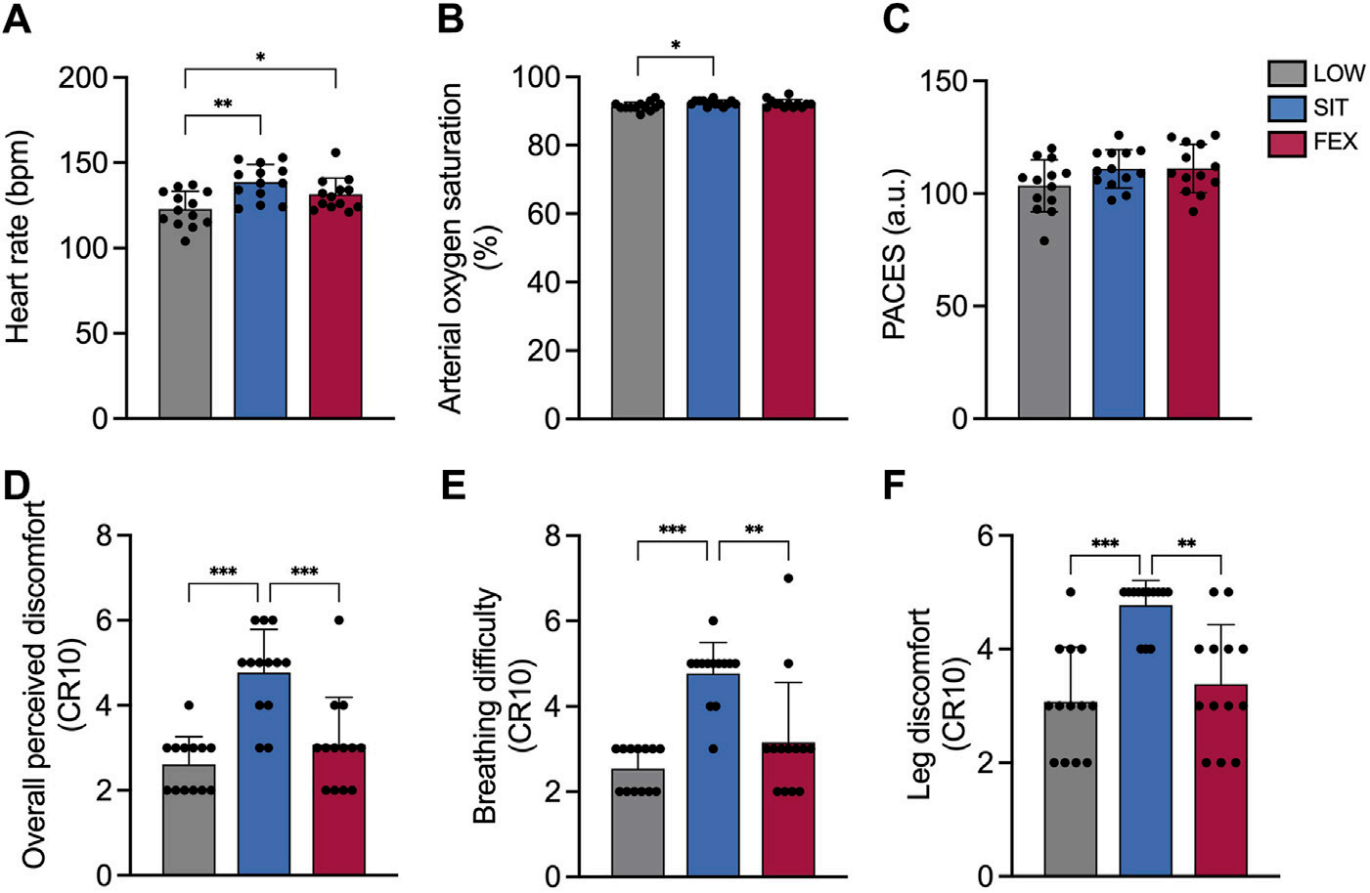
Heart rate **(A)**, arterial oxygen saturation (SpO_2_) **(B)**, physical activity enjoyment scale (PACES) **(C)**, overall perceived discomfort **(D)**, breathing difficulty **(E)** and leg discomfort **(F)** at three exercise modes under moderate hypoxia. Values are mean ± SD. *p < 0.05, **p < 0.01, ***p < 0.001 denotes a statistically difference between conditions.

There were significant differences between exercise conditions for HR (*p* < 0.001; η^2^_p_ = 0.53; Figure 4A) with higher HR responses in SIT compared to LOW (+13 ± 1%; *p* = 0.001; *g* = 1.46) and between FEX and LOW (+7 ± 7%; *p* = 0.021; *g* = 0.83). No significant differences were observed in HR between SIT and FEX (+5 ± 8%; *p* = 0.135; *g* = 0.68).

There were no significant differences between exercise conditions for SpO_2_ (*p* = 0.052; η^2^_p_ = 0.23; Figure 4B). No significant differences were observed between FEX and LOW (92% ± 1%; 91% ± 1%; *p* = 0.35; *g* = 0.60, respectively), as well as between FEX and SIT (92% ± 1%; 92% ± 1%, *p* > 0.99; *g* = 0.21, respectively).

#### Physical activity enjoyment scale (PACES)

There was no difference between exercise conditions for the physical activity enjoyment scale (*p* = 0.071; η^2^_p_ = 0.23 Figure 4C).

### Perceptual measures

There was a significant main effect of exercise conditions on overall perceived discomfort rating (*p* < 0.001; η^2^_p_ = 0.68; Figure 4D). Overall perceived discomfort rating was greater in SIT compared to both LOW and FEX (+82 ± 56%; *p* < 0.001; *g* = 2.45; +55 ± 9%; *p* < 0.001; *g* = 1.54, respectively). No significant differences were observed in overall perceived discomfort between FEX and LOW (+18 ± 71%; *p* = 0.570; *g* = 0.49). Significant differences were found between exercise conditions for perceived breathing difficulty (*p* < 0.001; η^2^_p_ = 0.68; Figure 4E) and perceived leg discomfort rating (*p* < 0.001; η^2^_p_ = 0.54; Figure 4F). Perceived breathing difficulty and leg discomfort rating were greater in SIT compared to both LOW and FEX (+88 ± 40%; *p* < 0.001; *g* = 3.43; +51 ± 48%; *p* = 0.003; *g* = 1.43; +55 ± 54%; *p* < 0.001; *g* = 2.21; +41 ± 58%; *p* = 0.001; *g* = 1.67, respectively). No significant difference was observed in either perceived breathing difficulty or leg discomfort rating in LOW compared to FEX (+24 ± 171%; +10 ± 9%; *p* > 0.05; *g* = 0.30–0.56, respectively).

## Discussion

The current study demonstrated that both low-intensity and sprint interval cycle exercise performed under moderate hypoxia (2000 m) improved post-exercise OGTT plasma glucose and insulin iAUC compared to control. Although HR and perceptual responses (i.e., overall perceived discomfort, perceived breathing difficulty, and leg discomfort) were higher in sprint interval cycle exercise, the PACES remained similar across different exercise modes. Collectively, we provide new information to indicate that significant improvements in acute blood glucose regulation can be attained with both low-intensity and sprint interval cycle exercise under moderate hypoxia in physically inactive adults with overweight.

Post-exercise plasma glucose and insulin have previously been demonstrated to be improved with low-intensity cycle exercise performed under moderate-to-high hypoxia (∼2000–3,000 m) in individuals with overweight (Tee et al., 2023b; Tee et al., 2023c) and type 2 diabetes (T2D) (Mackenzie et al., 2011; Mackenzie et al., 2012). In comparison, improvements in blood glucose responses following sprint interval training (Kon et al., 2015; Lei et al., 2022) or functional exercise (Ferrari et al., 2021) are less apparent, regardless of hypoxic levels. Our study is the first to directly compare different types of exercise under the same hypoxic (moderate) conditions as an adjuvant to exercise in male adults with overweight. Such knowledge can be practically relevant for individuals with overweight or obese and with time constraints to exercise (40 min for SIT vs. 60 min for LOW). Despite the acute nature of our current study, the findings provide an initial basis to demonstrate that adults with overweight, when engaging in low-intensity and sprint interval cycle exercise at moderate hypoxia, can promote significant improvements in post-exercise 2 h OGTT plasma glucose and insulin iAUC responses when compared to functional exercise in moderate hypoxia. While we did not obtain skeletal muscle samples to investigate the precise mechanisms underlying the enhancements in post-exercise glucose regulation induced by hypoxic conditioning, we speculate that the increased recruitment of muscle fibers during high-intensity exercise (Edgett et al., 2013) and the heightened physiological demands of exercising in hypoxia (Tee et al., 2023b) may contribute to overall greater improvements in plasma insulin iAUC. Additionally, it has been previously demonstrated that high-intensity exercise leads to a rapid depletion of muscle glycogen (MacDougall et al., 1977) which is crucial in the post-exercise enhancement of peripheral insulin sensitivity (Devlin et al., 1987).

In contrast to venous plasma glucose responses, no discernible differences in the post-exercise interstitial glucose response to the OGTT using CGM were observed regardless of exercise mode under moderate hypoxia (Tee et al., 2023c). Disparities between venous and interstitial blood glucose measures have been reported in previous studies (Cengiz and Tamborlane, 2009). These distinctions may be attributed to the time delay in which interstitial glucose levels appear in the interstitial fluid (Rebrin et al., 2010), as well as the differing intervals for readings (15 min for CGM compared to 30 min for venous blood). When assessing at 24-h CGM data, we noticed a slight decrease in the 2 h OGTT interstitial glucose levels and fewer spikes during SIT under moderate hypoxia compared to LOW and FEX, although these changes were not statistically significant. Of note, the 24-h macronutrients and energy intake during exercise day were not significantly different between different exercise modes. Together, our data suggest that SIT cycle exercise under moderate hypoxia appears to be more effective at influencing post-exercise plasma insulin levels compared to LOW cycle exercise and FEX. Whether such differential patterns in response between exercise modalities persist with longer intervention periods combining hypoxia and exercise remains an important area of investigation, particularly considering the beneficial effects of all three exercise modalities on blood glucose regulation under normoxic conditions (Bird and Hawley, 2016; Su et al., 2023).

Our current study also noted changes in physiological variables (e.g., heart rate and arterial oxygen saturation), as well as changes in the PACES, and perceptual responses that have significant implications for exercise adherence. As expected, HR significantly increased during SIT cycle exercise under acute exposure to moderate hypoxia. The PACES values did not show any statistical variances across the three exercise modes under moderate hypoxia. Perceptual responses (i.e., ratings of overall perceived discomfort, perceived breathing difficulty, and leg discomfort) were greater in SIT, suggesting the exercise effort is greater compared to other exercise modes. These results are in line with previous observations examining physiological and perceptual responses to different exercise intensity and hypoxia levels where greater exercise intensity or exposure to greater hypoxia increased the perceptual responses (Hobbins et al., 2021; Tee et al., 2023c). However, in the current study, for the same enjoyment responses, the SIT (i.e., 111 points and 40 min, respectively) and FEX (i.e., 111 points and 31 min, respectively) are more time-efficient than LOW (i.e., 103 points and 60 min, respectively). The measurement of time could be critical to clarify the varying levels of enjoyment as PACES were taken immediately post-exercise in the present study which is in line with the aforementioned study (Kong et al., 2021). Thus, from a perceptual standpoint, participants may enjoy doing SIT and FEX which is more fun and time efficient as compared to low-moderate cycle exercise for longer duration (Foster et al., 2015) but LOW and SIT cycle exercise remain superior for post-exercise glucose regulation.

To shed light on potential novel mechanisms that may mediate metabolic adaptations with hypoxic conditioning, we investigated the expression of selected c-miRNAs. It has been reported that miRNAs can be found in different body fluids such as plasma, serum, and urine and may thus be differentially regulated in response to different stimuli such as exercise and hypoxia (Chen et al., 2008; Hanke et al., 2010). Considering our primary outcome measure of blood glucose responses post-exercise and between exercise modes with moderate hypoxia, we specifically chose to investigate miRNAs that have been previously implicated as mediators of exercise-induced glucose regulation (miR-486-5p and miR-126-5p) (Aoi et al., 2013; Bao et al., 2018) or inflammation related to cellular responses with hypoxia (miR-21-5p) (Baggish et al., 2011; Bao et al., 2018). Indeed, previous work has shown decreased expression of circulating miR-486 with exercise (Aoi et al., 2013; Barber et al., 2019) which may play a role in dysregulated blood glucose response through its regulation of fatty acid synthase (Angeles and Hudkins, 2016). Similarly, the expression of c-miR-126 has also been shown to be altered with exercise (Barber et al., 2019; Eyileten et al., 2021) and concomitantly a potential biomarker for type-2 diabetes due to its interference with insulin signaling by targeting IRS1 and IRS2, resulting in insulin resistance (Nigi et al., 2018). Despite the aforementioned improvements in post-exercise OGTT plasma glucose with both LOW and SIT cycle exercise, the expression of c-miRs-486-5p, -126-5p, and -21-5p were unchanged post-exercise with all modalities performed under moderate hypoxia. It is possible our time-points of analysis (i.e., pre-, immediately post-exercise, and 24 h post-exercise) may have missed the temporal resolution for observing alterations in these c-miRNAs with combined exercise and hypoxia. Furthermore, it is plausible the observed enhancement in plasma glucose responses with SIT were more confined to the translational level of protein regulation such that these improvements were mediated largely by increases in Akt-mediated skeletal muscle GLUT4 protein expression (Camera et al., 2010; Soo et al., 2023). We also cannot rule out that the unchanged post-exercise responses observed in our selected c-mIRNAs may be due to the plasma and not the exosomal sample pool being analysed, with previous work highlighting differences in expression patterns of particular miRNAs following exercise between each sample pool (D’Souza et al., 2018). In this regard, there is a paucity of knowledge to date that has investigated the expression of c-miRNAs in response to exercise performed in moderate hypoxia. Further studies investigating the expression of a diverse range of c-miRNAs over an extended time course are warranted to provide new knowledge for the capacity of miRNAs to regulate improvements in blood glucose regulation with different exercise modes and hypoxic conditions.

We acknowledge several limitations of the present study. Firstly, exercise was not workload-matched, and thus any differences observed could be due to total workload rather than modality *per se*. In this regard, we wanted our exercise workloads to be reflective of their typical inherent “real-world” application. Another important limitation of our study was that we only recruited males with overweight and normal glycemia levels, thus the generalizability of the results to female and clinical cohorts may be limited. There is a paucity of research comparing metabolic responses between females and males to intermittent hypoxia interventions with the potential for sex-specific differences related to altered vulnerabilities to acute mountain sickness and the effects of hormonal- and/or menstrual-cycle phases in women requiring concerted consideration (Burtscher et al., 2023). Furthermore, our study did not include normoxic conditions compared to our previous work (Tee et al., 2023c) where different levels of hypoxia were investigated. However, our previous work reported that the effect of acute aerobic exercise combined with moderate hypoxia was most effective for improving post-exercise blood glucose regulation in adults with overweight (Tee et al., 2023c). Thus, we chose moderate hypoxia for the current study to compare with different modes of exercise on post-exercise blood glucose regulation. Lastly, while previous studies have reported a positive effect of acute exercise under normoxia on the expression of c-miRs-486-5p, -126-5p, and 21-5p (Aoi et al., 2013; Liu et al., 2014), the expression of these c-miRNAs may be more apparent with repeated exercise sessions and exposure to hypoxia over several weeks to months concurrent with more substantial changes in health markers (e.g., plasma glucose and insulin).

## Conclusion

In conclusion, an acute bout of LOW and SIT exercise in combination with moderate hypoxia improved post-exercise plasma insulin responses. These results suggest that SIT combined with moderate hypoxia can produce an additive effect on improving post-exercise plasma insulin. Furthermore, its time-efficient nature is also an important factor contributing to promoting SIT as a priority for exercise prescription by exercise physiologists and clinicians alike to promote metabolic adaptations.

## Data Availability

The original contributions presented in the study are included in the article/Supplementary Material further inquiries can be directed to the corresponding author.
