# Supplementary figures and images for: Effects of exercise modality combined with moderate hypoxia on blood glucose regulation in adults with overweight
Source: Front Physiol. 2024 Jun 5;15:1396108. doi: 10.3389/fphys.2024.1396108 (PMC11188384; doi:10.3389/fphys.2024.1396108)

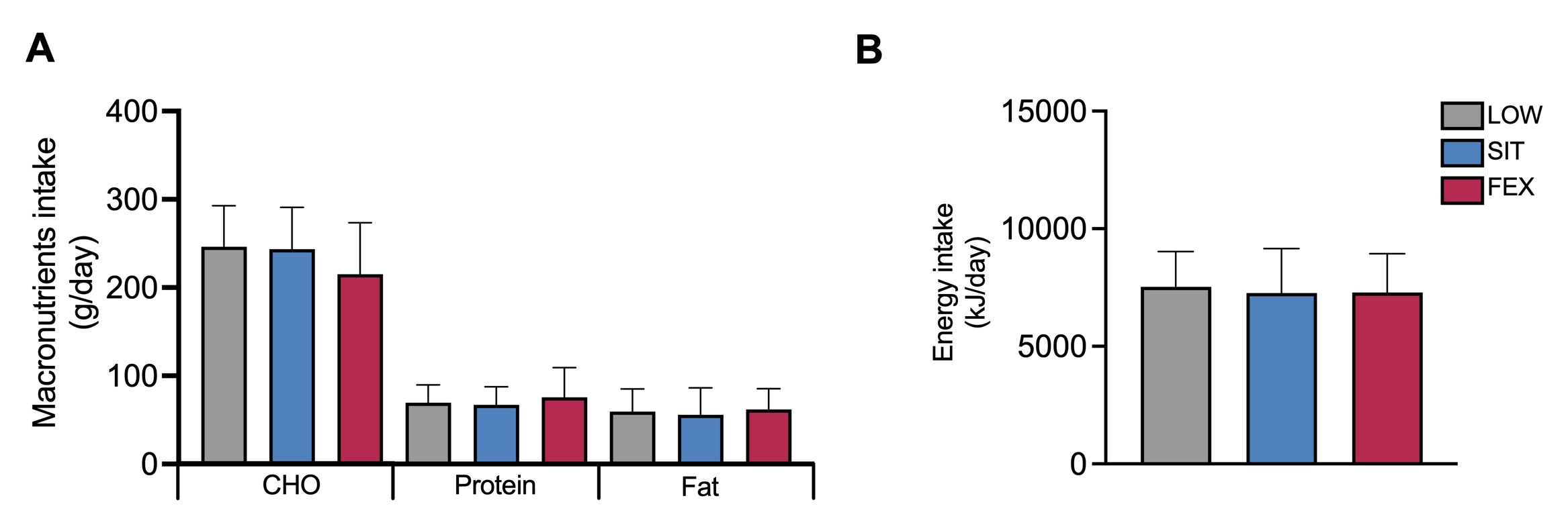

Supplement: Supplementary file 1 [file Image1.JPEG]
